# Redetermination of EuScO_3_

**DOI:** 10.1107/S1600536809001433

**Published:** 2009-01-17

**Authors:** Volker Kahlenberg, Dirk Maier, Boža Veličkov

**Affiliations:** a University of Innsbruck, Institute for Mineralogy and Petrography, Innrain 52, 6020 Innsbruck, Austria; b Leibniz-Institute for Crystal Growth, Max-Born-Strasse 2, 12489 Berlin, Germany

## Abstract

Single crystals of europium(III) scandate(III), with ideal formula EuScO_3_, were grown from the melt using the micro-pulling-down method. The title compound crystallizes in an ortho­rhom­bic distorted perovskite-type structure, where Eu occupies the eightfold coordinated *A* sites (site symmetry *m*) and Sc resides on the centres of corner-sharing [ScO_6_] octa­hedra (*B* sites with site symmetry 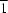). The structure of EuScO_3_ has been reported previously based on powder diffraction data [Liferovich & Mitchell (2004). *J. Solid State Chem.* **177**, 2188–2197]. The results of the current redetermination based on single-crystal diffraction data shows an improvement in the precision of the structral and geometric parameters and reveals a defect-type structure. Site-occupancy refinements indicate an Eu deficiency on the *A* site coupled with O defects on one of the two O-atom positions. The crystallochemical formula of the investigated sample may thus be written as ^*A*^(□_0.032_Eu_0.968_)^*B*^ScO_2.952_.

## Related literature

Details of the synthesis are described by Maier *et al.* (2007 ▶). Rietveld refinements on powders of *Ln*ScO_3_ with *Ln* = La^3+^ to Ho^3+^ are reported by Liferovich & Mitchell (2004 ▶). The crystal structures of the Dy, Gd, Sm and Nd members refined from single-crystal diffraction data have been recently provided by Veličkov *et al.* (2007 ▶). The crystal structure of the isotypic TbScO_3_ is described by Veličkov *et al.* (2008 ▶). Specific geometrical parameters have been calculated by means of the atomic coordinates following the concept of Zhao *et al.* (1993 ▶).

## Experimental

### 

#### Crystal data

Eu_0.968_ScO_2.952_
*M* *_r_* = 239.24
Orthorhombic, 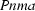

*a* = 5.7554 (7) Å
*b* = 7.9487 (10) Å
*c* = 5.5087 (6) Å
*V* = 252.01 (5) Å^3^
*Z* = 4
Mo *K*α radiation
μ = 26.28 mm^−1^
*T* = 293 (2) K
0.14 × 0.12 × 0.02 mm

#### Data collection

Stoe IPDS-2 diffractometer
Absorption correction: analytical (Alcock, 1970 ▶) *T*_min_ = 0.139, *T*_max_ = 0.397
2168 measured reflections
362 independent reflections
345 reflections with *I* > 2σ(*I*)
*R*_int_ = 0.055

#### Refinement

*R*[*F* ^2^ > 2σ(*F* ^2^)] = 0.021
*wR*(*F* ^2^) = 0.051
*S* = 1.27
362 reflections
31 parameters
1 restraint
Δρ_max_ = 1.40 e Å^−3^
Δρ_min_ = −0.84 e Å^−3^

### 

Data collection: *X-AREA* (Stoe & Cie, 2006 ▶); cell refinement: *X-AREA*; data reduction: *X-RED* (Stoe & Cie, 2006 ▶); program(s) used to solve structure: *SHELXS97* (Sheldrick, 2008 ▶); program(s) used to refine structure: *SHELXL97* (Sheldrick, 2008 ▶); molecular graphics: *ATOMS for Windows* (Dowty, 2004 ▶); software used to prepare material for publication: *SHELXL97*.

## Supplementary Material

Crystal structure: contains datablocks I, global. DOI: 10.1107/S1600536809001433/wm2214sup1.cif

Structure factors: contains datablocks I. DOI: 10.1107/S1600536809001433/wm2214Isup2.hkl

Additional supplementary materials:  crystallographic information; 3D view; checkCIF report

## Figures and Tables

**Table 1:** Selected bond lengths (Å)

Eu1—O1^i^	2.276 (5)
Eu1—O2^ii^	2.304 (3)
Eu1—O1^iii^	2.375 (5)
Eu1—O2^iv^	2.611 (3)
Eu1—O2^v^	2.845 (3)
Sc2—O2^ii^	2.094 (3)
Sc2—O2^vi^	2.107 (3)
Sc2—O1^vii^	2.1108 (16)

Symmetry codes: (i) 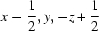; (ii) 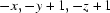; (iii) 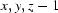; (iv) 
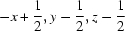
; (v) 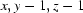; (vi) 
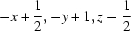
; (vii) 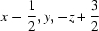.

**Fig. 1.**
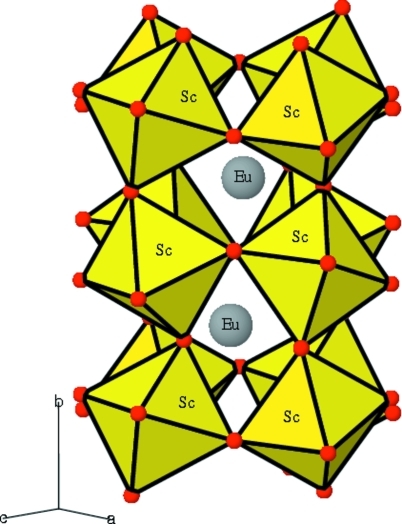
The orthorhombic perovskite structure of EuScO3 is characterized by a tilted corner sharing ScO6 framework incorporating the 8-fold coordinated Eu sites. The ScO6 octahedra are yellow, the Eu atoms are given in grey and the O atoms are presented in red.

**Fig. 2.**
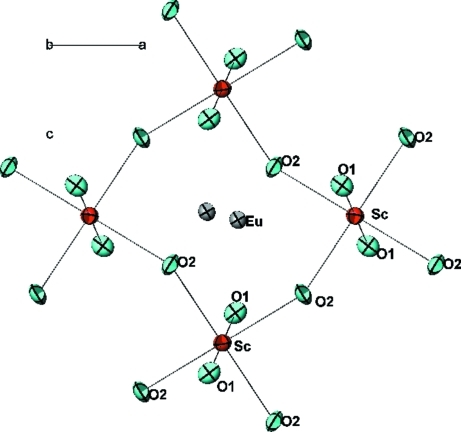
Projection of the EuScO3 structure along [010], showing the Eu atoms and the Sc coordination with displacement ellipsoids at the 80% probability level.

## supplementary crystallographic information

### Comment

Liferovich & Mitchell (2004) studied the crystal structure of lanthanoid scandates, including EuScO_3_, by Rietveld analysis from powder diffraction data. Crystallographic data of DyScO_3_, GdScO_3_, SmScO_3_ and NdScO_3_ obtained from single crystals were recently reported by Veličkov *et al.* (2007), and for TbScO_3_ by Veličkov *et al.* (2008). Based on these results it was possible to resolve disagreements concerning some structural characteristics and their dependence on the *Ln*-substitution. Whereas Liferovich & Mitchell (2004) observed no obvious continuous evolution, Veličkov *et al.* (2007) were able to show that the geometry and the distortion of the sites are linearly coupled with the size of the lanthanoid in the series from DyScO_3_ to NdScO_3_. With the structural refinement based on diffraction data collected from single crystalline EuScO_3_, the present results provide further data for this series.

The orthorhombic distorted perovskite structure for EuScO_3_ (Fig. 1) is confirmed from our refinements. Whereas the lattice parameters for EuScO_3_ compare well with the data of Liferovich & Mitchell (2004), the fractional atomic coordinates show deviations of up to 0.006, resulting in slightly different geometrical parameters. The *A*-site is occupied by Eu in an eightfold coordination and has an average bond length of ^[8]^<*A*—O> = 2.521 Å with a polyhedral bond length distortion of *^A^*Δ_8_ = 7.98×10^-3^ (Δn = 1/n Σ{(r_i_-r)/r^2^). The *B*-site shows bond lengths typical for octahedrally coordinated scandium (<*B*—O> = 2.104 Å) and is rather distorted with *^B^*Δ_6_ = 0.015×10^-3^ and a bond angle variance of δ = 2.61°. The tilting of the corner sharing octahedra calculated after Zhao *et al.* (1993) are θ = 19.70° in [110] and Ø = 12.66° in [001] directions. From our data we can establish linear trends for the crystallochemical parameters from DyScO_3_ to NdScO_3_ depending on the *Ln*-substitution.

### Experimental

An EuScO_3_ fiber was grown using a micro-pulling-down apparatus with induction heating (Maier *et al.*, 2007). The starting material was prepared from 4 N Eu_2_O_3_ and Sc_2_O_3_ powders by grinding a total weight of 5 g in a plastic mortar and pressing the mixture into pellets. An 5 ml iridium crucible with 500 mg of the starting material was placed on an iridium after-heater and heated by the inductor coil of a 10 kW rf Generator. The crucible after-heater arrangement was surrounded by a zirconia fiber tube and a high-purity alumina tube for thermal insulation. The experiments were carried out in a vacuum-tight steel chamber. It was evacuated before each experiment to 5 x10^-3^ mbar and filled with 5 N argon gas. Subsequently, a constant flow of about 900 ml/min was kept. During growth the height of the molten zone and consequently the diameter of the fiber (~1 mm) was carefully controlled by manually increasing or decreasing the rf-power. Several starting compositions with different Eu to Sc ratios were tested, resulting in the optimal batch with 47.5 mol% Eu_2_O_3_ and 52.5 mol% Sc_2_O_3_.

The grown single-crystal was colourless. A part of the single-crystal fiber was crushed and the irregular shaped fragments were screened using a polarizing light microscope to find a sample of good optical quality for the diffraction experiments.

### Refinement

Site occupation refinements indicated deviations from full occupancy on the Eu1 (*A*-site) and the O2 sites. For the final refinement cycle a constraint ensuring charge neutrality was included. The crystallochemical formula of the investigated sample can thus be written as *^A^*(□_0.032_Eu_0.968_)*^B^*ScO_2.952_. The highest peak and deepest hole of the difference Fourier map are located 0.84 Å and 1.42 Å away from the Eu1 position. In contrast to the previous Rietveld refinement, where the *Pbnm* setting of space group no. 62 was chosen, the standard setting *Pnma* was used for the present redetermination.

### Crystal data

**Table:**

Eu_0.968_ScO_2.952_	*F*(000) = 422.4
*M**_r_* = 239.24	*D*_x_ = 6.307 Mg m^−^^3^
Orthorhombic, *P**n**m**a*	Mo *K*α radiation, λ = 0.71073 Å
Hall symbol: -P 2ac 2n	Cell parameters from 2218 reflections
*a* = 5.7554 (7) Å	θ = 2.6–29.2°
*b* = 7.9487 (10) Å	µ = 26.28 mm^−^^1^
*c* = 5.5087 (6) Å	*T* = 293 K
*V* = 252.01 (5) Å^3^	Platy fragment, colourless
*Z* = 4	0.14 × 0.12 × 0.02 mm

### Data collection

**Table:**

Stoe IPDS-2 diffractometer	362 independent reflections
Radiation source: fine-focus sealed tube	345 reflections with *I* > 2σ(*I*)
graphite	*R*_int_ = 0.055
Detector resolution: 6.67 pixels mm^-1^	θ_max_ = 29.1°, θ_min_ = 4.5°
ω scans	*h* = −7→7
Absorption correction: analytical (Alcock, 1970)	*k* = −10→9
*T*_min_ = 0.139, *T*_max_ = 0.397	*l* = −7→7
2168 measured reflections	

### Refinement

**Table:**

Refinement on *F*^2^	Primary atom site location: structure-invariant direct methods
Least-squares matrix: full	Secondary atom site location: difference Fourier map
*R*[*F*^2^ > 2σ(*F*^2^)] = 0.021	*w* = 1/[σ^2^(*F*_o_^2^) + (0.0211*P*)^2^ + 0.9412*P*] where *P* = (*F*_o_^2^ + 2*F*_c_^2^)/3
*wR*(*F*^2^) = 0.051	(Δ/σ)_max_ = 0.011
*S* = 1.27	Δρ_max_ = 1.40 e Å^−^^3^
362 reflections	Δρ_min_ = −0.84 e Å^−^^3^
31 parameters	Extinction correction: *SHELXL97* (Sheldrick, 2008), Fc^*^=kFc[1+0.001xFc^2^λ^3^/sin(2θ)]^-1/4^
1 restraint	Extinction coefficient: 0.113 (5)

### Special details

**Table:**

Geometry. All e.s.d.'s (except the e.s.d. in the dihedral angle between two l.s. planes) are estimated using the full covariance matrix. The cell e.s.d.'s are taken into account individually in the estimation of e.s.d.'s in distances, angles and torsion angles; correlations between e.s.d.'s in cell parameters are only used when they are defined by crystal symmetry. An approximate (isotropic) treatment of cell e.s.d.'s is used for estimating e.s.d.'s involving l.s. planes.
Refinement. Refinement of *F*^2^ against ALL reflections. The weighted *R*-factor *wR* and goodness of fit *S* are based on *F*^2^, conventional *R*-factors *R* are based on *F*, with *F* set to zero for negative *F*^2^. The threshold expression of *F*^2^ > σ(*F*^2^) is used only for calculating *R*-factors(gt) *etc*. and is not relevant to the choice of reflections for refinement. *R*-factors based on *F*^2^ are statistically about twice as large as those based on *F*, and *R*- factors based on ALL data will be even larger.

### Fractional atomic coordinates and isotropic or equivalent isotropic displacement parameters (Å^2^)

**Table:**

	*x*	*y*	*z*	*U*_iso_*/*U*_eq_	Occ. (<1)
Eu1	0.05854 (5)	0.25	0.01589 (6)	0.0090 (2)	0.9677 (13)
Sc2	0	0	0.5	0.0078 (3)	
O1	0.4506 (8)	0.25	0.8815 (9)	0.0111 (9)	
O2	0.1954 (6)	0.9378 (4)	0.8078 (6)	0.0112 (7)	0.9758 (10)

### Atomic displacement parameters (Å^2^)

**Table:**

	*U*^11^	*U*^22^	*U*^33^	*U*^12^	*U*^13^	*U*^23^
Eu1	0.0070 (3)	0.0102 (3)	0.0098 (3)	0	0.00051 (11)	0
Sc2	0.0064 (6)	0.0079 (6)	0.0090 (6)	0.0006 (6)	0.0000 (3)	0.0000 (3)
O1	0.011 (2)	0.008 (2)	0.014 (2)	0	−0.0025 (17)	0
O2	0.0096 (16)	0.0121 (17)	0.0119 (14)	−0.0019 (12)	−0.0018 (11)	0.0005 (12)

### Geometric parameters (Å, °)

**Table:**

Eu1—O1^i^	2.276 (5)	Sc2—O2^ii^	2.094 (3)
Eu1—O2^ii^	2.304 (3)	Sc2—O2^xii^	2.094 (3)
Eu1—O2^iii^	2.304 (3)	Sc2—O2^vi^	2.107 (3)
Eu1—O1^iv^	2.375 (5)	Sc2—O2^xiii^	2.107 (3)
Eu1—O2^v^	2.611 (3)	Sc2—O1^xiv^	2.1108 (16)
Eu1—O2^vi^	2.611 (3)	Sc2—O1^x^	2.1108 (16)
Eu1—O2^vii^	2.845 (3)	Sc2—Eu1^xv^	3.2268 (4)
Eu1—O2^viii^	2.845 (3)	Sc2—Eu1^i^	3.2268 (4)
Eu1—Sc2^ix^	3.2268 (4)	Sc2—Eu1^xvi^	3.3428 (4)
Eu1—Sc2^x^	3.2268 (4)	Sc2—Eu1^xvii^	3.4841 (4)
Eu1—Sc2^xi^	3.3428 (4)	Sc2—Eu1^xviii^	3.4841 (4)
Eu1—Sc2	3.3428 (4)		
			
O1^i^—Eu1—O2^ii^	103.43 (12)	O2^xii^—Sc2—O2^vi^	90.88 (6)
O1^i^—Eu1—O2^iii^	103.43 (12)	O2^ii^—Sc2—O2^xiii^	90.88 (6)
O2^ii^—Eu1—O2^iii^	80.77 (17)	O2^xii^—Sc2—O2^xiii^	89.12 (6)
O1^i^—Eu1—O1^iv^	87.69 (11)	O2^vi^—Sc2—O2^xiii^	180
O2^ii^—Eu1—O1^iv^	137.24 (9)	O2^ii^—Sc2—O1^xiv^	87.46 (16)
O2^iii^—Eu1—O1^iv^	137.24 (9)	O2^xii^—Sc2—O1^xiv^	92.54 (16)
O1^i^—Eu1—O2^v^	137.77 (9)	O2^vi^—Sc2—O1^xiv^	92.67 (15)
O2^ii^—Eu1—O2^v^	117.06 (6)	O2^xiii^—Sc2—O1^xiv^	87.33 (15)
O2^iii^—Eu1—O2^v^	73.38 (8)	O2^ii^—Sc2—O1^x^	92.54 (16)
O1^iv^—Eu1—O2^v^	71.14 (12)	O2^xii^—Sc2—O1^x^	87.46 (16)
O1^i^—Eu1—O2^vi^	137.77 (9)	O2^vi^—Sc2—O1^x^	87.33 (15)
O2^ii^—Eu1—O2^vi^	73.38 (8)	O2^xiii^—Sc2—O1^x^	92.67 (15)
O2^iii^—Eu1—O2^vi^	117.06 (6)	O1^xiv^—Sc2—O1^x^	180
O1^iv^—Eu1—O2^vi^	71.14 (12)	Sc2^xix^—O1—Sc2^xv^	140.6 (2)
O2^v^—Eu1—O2^vi^	69.74 (15)	Sc2^xix^—O1—Eu1^xx^	105.10 (13)
O1^i^—Eu1—O2^vii^	71.82 (8)	Sc2^xv^—O1—Eu1^xx^	105.10 (13)
O2^ii^—Eu1—O2^vii^	77.31 (12)	Sc2^xix^—O1—Eu1^xviii^	91.82 (14)
O2^iii^—Eu1—O2^vii^	155.61 (9)	Sc2^xv^—O1—Eu1^xviii^	91.82 (14)
O1^iv^—Eu1—O2^vii^	67.12 (8)	Eu1^xx^—O1—Eu1^xviii^	124.0 (2)
O2^v^—Eu1—O2^vii^	126.63 (6)	Sc2^xxi^—O2—Sc2^xxii^	143.00 (18)
O2^vi^—Eu1—O2^vii^	66.38 (5)	Sc2^xxi^—O2—Eu1^ii^	98.83 (13)
O1^i^—Eu1—O2^viii^	71.82 (8)	Sc2^xxii^—O2—Eu1^ii^	117.90 (14)
O2^ii^—Eu1—O2^viii^	155.61 (9)	Sc2^xxi^—O2—Eu1^xxii^	85.85 (11)
O2^iii^—Eu1—O2^viii^	77.31 (12)	Sc2^xxii^—O2—Eu1^xxii^	89.57 (12)
O1^iv^—Eu1—O2^viii^	67.12 (8)	Eu1^ii^—O2—Eu1^xxii^	103.48 (13)
O2^v^—Eu1—O2^viii^	66.38 (5)	Sc2^xxi^—O2—Eu1^xxiii^	88.37 (12)
O2^vi^—Eu1—O2^viii^	126.63 (6)	Sc2^xxii^—O2—Eu1^xxiii^	79.82 (10)
O2^vii^—Eu1—O2^viii^	121.45 (13)	Eu1^ii^—O2—Eu1^xxiii^	102.69 (12)
O2^ii^—Sc2—O2^xii^	180	Eu1^xxii^—O2—Eu1^xxiii^	153.77 (14)
O2^ii^—Sc2—O2^vi^	89.12 (6)		

Symmetry codes: (i) *x*−1/2, *y*, −*z*+1/2; (ii) −*x*, −*y*+1, −*z*+1; (iii) −*x*, *y*−1/2, −*z*+1; (iv) *x*, *y*, *z*−1; (v) −*x*+1/2, *y*−1/2, *z*−1/2; (vi) −*x*+1/2, −*y*+1, *z*−1/2; (vii) *x*, *y*−1, *z*−1; (viii) *x*, −*y*+3/2, *z*−1; (ix) *x*+1/2, −*y*+1/2, −*z*+1/2; (x) −*x*+1/2, −*y*, *z*−1/2; (xi) −*x*, *y*+1/2, −*z*+1; (xii) *x*, *y*−1, *z*; (xiii) *x*−1/2, *y*−1, −*z*+3/2; (xiv) *x*−1/2, *y*, −*z*+3/2; (xv) −*x*+1/2, −*y*, *z*+1/2; (xvi) −*x*, −*y*, −*z*+1; (xvii) −*x*, −*y*, −*z*; (xviii) *x*, *y*, *z*+1; (xix) *x*+1/2, −*y*+1/2, −*z*+3/2; (xx) *x*+1/2, *y*, −*z*+1/2; (xxi) *x*, *y*+1, *z*; (xxii) −*x*+1/2, −*y*+1, *z*+1/2; (xxiii) *x*, *y*+1, *z*+1.
